# Methylation of *NR3C1* is related to maternal PTSD, parenting stress and maternal medial prefrontal cortical activity in response to child separation among mothers with histories of violence exposure

**DOI:** 10.3389/fpsyg.2015.00690

**Published:** 2015-05-29

**Authors:** Daniel S. Schechter, Dominik A. Moser, Ariane Paoloni-Giacobino, Ludwig Stenz, Marianne Gex-Fabry, Tatjana Aue, Wafae Adouan, María I. Cordero, Francesca Suardi, Aurelia Manini, Ana Sancho Rossignol, Gaëlle Merminod, Francois Ansermet, Alexandre G. Dayer, Sandra Rusconi Serpa

**Affiliations:** ^1^Geneva Early Childhood Stress Project, Department of Child and Adolescent Psychiatry, Faculty of Medicine, University of Geneva HospitalsGeneva, Switzerland; ^2^Division of Developmental Neuroscience, Department of Psychiatry, Columbia University College of Physicians and SurgeonsNew York, NY, USA; ^3^Department of Genetic Medicine and Development, Faculty of Medicine, University of GenevaGeneva, Switzerland; ^4^Department of Psychiatry, Faculty of Medicine, University of Geneva HospitalsGeneva, Switzerland; ^5^Swiss Center for Affective Sciences, University of GenevaGeneva, Switzerland; ^6^Division of Experimental Psychology and Neuropsychology, Department of Psychology, University of BernBern, Switzerland; ^7^Faculty of Health, Psychology and Social Care, Manchester Metropolitan UniversityManchester, UK

**Keywords:** PTSD, glucocorticoid receptor (*NR3c1*), fMRI, parenting, interpersonal violence, early life stress, epigenetics, methylation

## Abstract

Prior research has shown that mothers with Interpersonal violence-related posttraumatic stress disorder (IPV-PTSD) report greater difficulty in parenting their toddlers. Relative to their frequent early exposure to violence and maltreatment, these mothers display dysregulation of their hypothalamic pituitary adrenal axis (HPA-axis), characterized by hypocortisolism. Considering methylation of the promoter region of the glucocorticoid receptor gene *NR3C1* as a marker for HPA-axis functioning, with less methylation likely being associated with less circulating cortisol, the present study tested the hypothesis that the degree of methylation of this gene would be negatively correlated with maternal IPV-PTSD severity and parenting stress, and positively correlated with medial prefrontal cortical (mPFC) activity in response to video-stimuli of stressful versus non-stressful mother–child interactions. Following a mental health assessment, 45 mothers and their children (ages 12–42 months) participated in a behavioral protocol involving free-play and laboratory stressors such as mother–child separation. Maternal DNA was extracted from saliva. Interactive behavior was rated on the CARE-Index. During subsequent fMRI scanning, mothers were shown films of free-play and separation drawn from this protocol. Maternal PTSD severity and parenting stress were negatively correlated with the mean percentage of methylation of *NR3C1*. Maternal mPFC activity in response to video-stimuli of mother–child separation versus play correlated positively to *NR3C1* methylation, and negatively to maternal IPV-PTSD and parenting stress. Among interactive behavior variables, child cooperativeness in play was positively correlated with *NR3C1* methylation. Thus, the present study is the first published report to our knowledge, suggesting convergence of behavioral, epigenetic, and neuroimaging data that form a psychobiological signature of parenting-risk in the context of early life stress and PTSD.

## Introduction

Posttraumatic stress disorder (PTSD) is a form of psychopathology that is typically characterized by dysregulation of the hypothalamic pituitary adrenal (HPA) axis. In the face of perceived danger that does not extinguish over time, the HPA axis must produce circulating glucocorticoids in order to mobilize energy to fuel the organism’s fight or flight response to traumatic reminders and to regulate its overall stress response. It is known that chronic HPA axis dysregulation that leads to excessive exposure of developing brain areas to glucocorticoids is deleterious to the organism (Sorrells and Sapolsky, 2007).

Hypothalamic pituitary adrenal axis dysregulation can lead to: (a) excessive glucocorticoid secretion in response to stressors (i.e., hypercortisolism), (b) lack of circulating cortisol and diminished reactivity to stressors (i.e., hypocortisolism), as a consequence of the system’s depletion or as a protective adaptation to a persistently threatening environment. Both of these possible outcomes are likely determined by multiple factors such as the nature of the traumatogenic event, its onset, duration, and chronicity as well as the onset, duration, and chronicity of the PTSD and its comorbidity.

Several studies have shown that PTSD following acute “single-event” type trauma such as car accidents or natural catastrophes is generally associated with elevated baseline cortisol and high reactivity to trauma-evocative stimuli in the laboratory (Pervanidou et al., 2007). PTSD that develops in the wake of repeated childhood maltreatment and domestic violence, or adult exposures to domestic violence or combat, has been associated with hypocortisolism (Elzinga et al., 2008). This hypocortisolism is observed at baseline, over the diurnal curve and in response to trauma-evocative stimuli in the laboratory (Daskalakis et al., 2013). Whether there are sensitive periods during development during which individuals are more vulnerable to stress-induced HPA axis dysregulation in one direction or the other remains to be established. With this in mind, Yehuda et al. (2000, 2007) have suggested that hypocortisolism is linked to maternal PTSD and transmission of vulnerability for PTSD via fetal programming that renders the infant vulnerable himself to develop PTSD. This hypothesis has stimulated interest in examining the possibility of non-genomic (i.e., epigenetic) transmission of adaptation to traumatogenic environments across generations (Yehuda et al., 2014a).

Researchers have begun to turn their attention to epigenetic factors involved in HPA axis response and adaptation to stressors by studying the methylation status of genes involved in HPA regulation. These genes include the promoter region of *NR3C1* gene coding for the glucocorticoid receptor. Greater methylation has been linked to decreased *NR3C1* mRNA transcription and protein levels in the hippocampus, a key structure involved in decreasing stress-induced HPA axis activation and, peripherally, in blood lymphocytes and saliva (McGowan et al., 2009; Tyrka et al., 2012; Weder et al., 2014).

Two papers involving a sample with a primary diagnosis of combat-related PTSD have demonstrated an inverse association between the severity of PTSD symptoms and the degree of methylation of the promoter region of the glucocorticoid receptor *NR3C1* (Yehuda et al., 2013, 2014b). In contrast, a study focused on women with histories of maltreatment and borderline personality disorder found that a history of maltreatment, and particularly of sexual and emotional abuse, was associated with greater methylation of the promoter region of the *NR3C1* gene (Perroud et al., 2011). These findings have been replicated in at least two subsequent studies with a focus on different forms of adverse events such as childhood physical abuse (Tyrka et al., 2012; Romens et al., 2014).

These studies that have looked at adverse early life events such as childhood physical and sexual abuse have focused on diverse samples including healthy individuals (Tyrka et al., 2012) and women with borderline personality (Perroud et al., 2011) but have not systematically looked at the effect of possible PTSD and how PTSD might be related to methylation patterns. Adults who have experienced childhood physical and sexual abuse are, upon exposure to interpersonal violent (IPV) events during adulthood, more likely to develop PTSD (Breslau et al., 2014). It is thus important to examine the relationship of *NR3C1* methylation to adult IPV-PTSD.

Relatively few studies have examined the impact of maternal PTSD. Studies that have focused on maternal IPV-PTSD have nevertheless shown a number of converging findings. Namely, maternal PTSD is associated with less sensitive maternal behavior that reflects more maternal avoidance and decreased responsiveness to child social bids (Cohen et al., 2008; Schechter et al., 2008, 2010). Maternal IPV-PTSD severity and atypical maternal behavior were both shown to be associated with low salivary cortisol baselines and blunted stress reactivity (Schechter et al., 2004; Crockett et al., 2013).

A study of maternal neural response to silent video stimuli depicting child separation as a stressful condition versus quiet play as a non-stressful control-condition showed decreased medial prefrontal cortex (mPFC) activity and increased entorhinal cortex activity among IPV-PTSD versus non-PTSD mothers (Schechter et al., 2012). Moreover, the severity of maternal PTSD has been associated with young children’s dysregulated aggression and increased anxiety, social withdrawal, and avoidance of interpersonal conflict in play following from story-stem completion (Schechter et al., 2007). Yet no published paper to our knowledge has heretofore demonstrated associations between maternal trauma and related psychopathology, HPA axis dysregulation as marked by altered methylation of the *NR3C1* gene, and maternal-child behavior, as related to neural activity in response to parenting stress -relevant stimuli.

We hypothesized that *NR3C1* methylation would inversely correlate to IPV-PTSD severity and would positively correlate to degree of activity in brain regions implicated in emotion regulation, such as the mPFC. We further hypothesized that maternal PTSD severity, *NR3C1* methylation, and maternal neural activity in response to separation-stress stimuli would predict greater parenting stress and disturbance of mother–child interactive behavior as measured by maternal sensitivity and child cooperativeness during play.

## Materials and Methods

### Participants and Procedures

The study protocol was approved by the institutional review board of the University of Geneva Hospitals and in accordance with the Helsinki Declaration (World Medical Association, 1999).

Inclusion and exclusion criteria were as follows: biological mothers were included in the study if they had lived with their child for the majority of the child’s life since birth. Due to physiological measurements taken that could be altered by hormonal changes associated with pregnancy and breastfeeding, women who were in either category were not accepted into the study until 30 days after they had stopped breast-feeding. Children were included in the study if they were 12–42 months of age at the time of scheduled mother–child behavioral observations. Women and their children were recruited by flyers posted at the University of Geneva Hospitals and Faculties of Medicine and Psychology as well as at community agencies such as neighborhood centers, daycares, and schools. In order to ensure adequate representation of women with violence exposure and PTSD, recruitment efforts were targeted to programs serving women seeking professional help or shelter following domestic violence exposure. All comers were screened. Fathers and other romantic partners of mothers were not seen in the study given concerns over safety and maintenance of trust for women who had experienced partner violence. Thus data about fathers were obtained by maternal report rather than from the fathers themselves.

Within 1 month after the screening visit, participants completed two videotaped visits over the ensuing 1–2 months period. During the screening visit, following informed consent, mothers were given a socio-demographic and life-events interview followed by several self-report questionnaires. During the next visit, mothers were interviewed without their child present, with a focus on the mother’s mental representations of her child and relationship with her child, an elaboration of her traumatic life-events, followed by structured diagnostic interviews and a series of dimensional measures. Then, 1–2 weeks later, mothers were asked to bring their child to the lab for a mother–child interaction procedure otherwise known as the “Modified Crowell Procedure” (Zeanah et al., 2000). This procedure involves free play, separation–reunion, structured play, repeated separation–reunion and exposure to novelty (i.e., the entrance of a masked clown and noise-making, robotic toys such as a dinosaur and jumping spider toy). This mother–child interaction procedure was followed by administration of measures focusing on the child’s life events, psychopathology, and social–emotional development. Saliva samples were taken from mothers and children for measurement of cortisol and DNA extraction (as described in more detail below) prior to the Modified Crowell Procedure, immediately afterward and then 30 and 60 min later. After each of these visits, mothers received 50 Swiss francs along with a small book or toy for their child following the parent–child visit.

All mothers were invited to complete the MRI 2–4 weeks after the clinical interview and mother–child observations. Mothers who consented and were eligible for MRI were scanned at the hospital-based neuroimaging center. There they first completed routine pre-scanning questionnaires. After a clinician and neuroimaging specialist-guided orientation to the MRI scanner and scanning process, mothers participated in the fMRI protocol as described below, followed by a post-scanning semi-structured clinical interview to probe mothers’ experiences in the scanner (Schechter et al., 2012).

Out of 70 mothers and children who were screened and provided informed consent, four mothers had a full-PTSD diagnosis or clinically significant symptoms (subthreshold) primarily due to a non-IPV traumatic event (i.e., medical–surgical event, accident, natural disaster, etc.) and were thus excluded from the present analyses. Thus, 66 French-speaking mothers (ages 18–45 years) and children (ages 12–42 months) participated. For a subset of 49 mothers, datasets including successful DNA extraction from saliva and maternal sensitivity coding were available. Out of these 49, 4 participants were excluded due to lack of sufficient DNA quality to permit acquisition of *NR3C1* methylation data. This left 45 participants for data analysis, who were identified as “IPV-PTSD mothers” (*n* = 28) or “non-PTSD controls” (*n* = 17), as described below.

### Measures

#### Socio-Demographic Variables and Life-Events

During the screening session we conducted an interview with the mothers using the Geneva Socio-demographic Questionnaire (GSQ; Sancho Rossignol et al., unpublished) which was adapted from the Structured Clinical Interview for the DSM-IV (First et al., 1995) and developed for the present study in order to obtain a detailed overview of the parents’ socioeconomic status, characteristics, and history of the mother–partner relationships, and exposure to stressful life-events [i.e., interpersonal violence (IPV), substance abuse, economic difficulties, immigration, physical and mental health problems and interventions, and child protective and judicial services involvement]. The family socio-economic status (SES) was calculated using the Largo Index (Largo et al., 1989), which is a well-validated SES index used in pediatric research in Switzerland that takes into account both parental educational attainment and occupational status.

#### IPV and Other Traumatic Life Events

History of traumatic events during childhood was assessed via the Brief Physical and Sexual Abuse Questionnaire (BPSAQ; Marshall et al., 1998), and supplemented for other events during adulthood with the Traumatic Life Events Questionnaire (TLEQ). The TLEQ assesses 22 life events that could fulfill the “A-Criterion” for the DSM-IV diagnosis of PTSD. The TLEQ shows stability and convergent validity across various studies (Kubany et al., 2000). Twelve items that asked for the same events as the BPSAQ were eliminated from the TLEQ. Scoring of the BPSAQ was undertaken as described in a previous paper by the first author (Schechter et al., 2005). The severity of physical violence of the mother’s partner and herself in the context of her adult romantic relationships was measured via the Conflicts Tactics Scale 2 Short Version (CTS2; Straus and Douglas, 2004). This well-validated measure consists of 20 items that ask about tactics used by the participant’s partner and herself in order to resolve relational conflict including physical aggression along a seven-point scale.

#### Maternal Psychopathology

During the first videotaped interview IPV-exposed and non-IPV-exposed mothers underwent a variety of psychometric evaluations including the Clinician administered PTSD scale (CAPS; Blake et al., 1995) to assess lifetime PTSD and the Post-traumatic Symptom Checklist -Short Version (PCL-S) to assess current PTSD symptoms (Weathers et al., 2001). Participants with no IPV and no PTSD symptoms on both measures were coded as having the minimum score of 16. Participants with IPV exposure but no PTSD symptoms were coded as having 17.

For categorical analyses, mothers met criteria for violence-related PTSD if their DSM-IV A-criterion trauma was of a violent nature (i.e., due to child physical or sexual abuse or family violence exposure and/or adult physical or sexual assault).

Three groups of participants were then identified based on whether the participant fully met DSM-IV diagnostic criteria for lifetime PTSD on the CAPS and current PTSD with a score of 40 or more on the PCL-S for symptoms occurring within the past month (*n* = 17; Blanchard et al., 1996). A second group that met full criteria for lifetime diagnosis on the CAPS and yet had significant clinical symptoms of PTSD without meeting full diagnostic criteria for current PTSD (PCL-S score of 31–40) was identified as “subthreshold” for current PTSD (*n* = 11). Participants who did not meet criteria on either measure were considered to be “non-PTSD” controls (*n* = 17). Of note, many non-PTSD controls as shown in **Table 1B** were also exposed to IPV and had some PTSD symptoms that were not clinically significant enough to put them into the subthreshold group. For group comparison, full-criteria for diagnosis and subthreshold groups were combined into a clinical IPV-PTSD group referred to in this paper as “IPV-PTSD mothers” (*n* = 28) to be compared to “non-PTSD controls” (*n* = 17).

#### Parenting Stress

Parenting Stress was measured via the Parenting Stress Index—Short Form (PSI-SF; Abidin, 1995). This score includes items related to distress that parents feel in relation to their role as a parent and in light of other personal stressors, as well as parent–child relationship dysfunction, and child behavior that poses difficulty to parents. The PSI-SF has 36 items and each item is assessed on a five-point Likert scale, from 1 (strongly disagree) to 5 (strongly agree). It is a standardized instrument with a validated French translation. The PSI-SF shows high internal consistency (Cronbach’s alpha = 0.92; Abidin, 1995).

#### Observed Maternal and Child Behavior

Maternal sensitivity and child cooperativeness were measured via structured behavioral observations during 5 min of mother–child play. Two blind raters who were psychologists trained to reliability on the CARE-Index (Crittenden, unpublished) coded the maternal and child behaviors. The coding procedure focused these raters’ attention on seven aspects of maternal and child behavior, concerned with affect (facial expression, vocal expression, position and body contact, expression of affection), and “cognition” (i.e., temporal order and interpersonal contingency such as pacing of turns, control of the activity, and developmental appropriateness of the activity). Each aspect of behavior was evaluated separately. The scores were then summed to generate the maternal sensitivity and child cooperativeness scale scores. Both scores have a range from 0 to 14, with zero being dangerously insensitive/uncooperative to point of disruptive or unengaged, 7 being normally sensitive/cooperative, and 14 being outstandingly sensitive/cooperative. Inter-rater reliability was excellent (ICC = 0.86). The Infant and Toddler versions of the CARE-Index are well-validated (Farnfield et al., 2010; Kunster et al., 2010).

#### fMRI Stimuli, Study Design, and Data Acquisition

fMRI stimuli were drawn from mother–child interaction sequences of free-play and separation embedded within the 25-min mother–child interaction (i.e., Modified Crowell Procedure) as described above. A research assistant who was blind to case-control status among mothers’ own children selected the silent excerpts for the fMRI stimulus of play and separation: mothers viewed the play-excerpt that was observed to show the most joy and reciprocally the separation excerpt that showed the strongest child emotional response in terms of negative emotion and distressed behavior.

Mothers viewed six different, silent, 30-s video-excerpts of three children, each during the two conditions (separation and play): (1) own child, (2) unfamiliar boy, and (3) unfamiliar girl. The unfamiliar children conditions were obtained by filming two mothers and their children who did not participate in the study.

The fMRI study design consisted of two runs, each lasting 15 min, and each containing three blocks during which mothers viewed all six 30-s video excerpts, in a pseudorandom order, counterbalanced within and across runs. Thus, mothers viewed each of the six 30-s film clips six times. Each sequence was preceded by a 2-s white board either saying “mother and child play” or “child during separation.” In order to maximize the likelihood that participants would actually watch the stimuli, we also filmed participants’ eye gaze with an eye tracker (Eye-Trac 6, Applied Science Laboratories, Bedford, MA, USA) during the scan. Detailed image acquisition and pre-processing are described in the Supplementary Materials. After the MRI visit, mothers received 200 Swiss francs.

Out of the 45 mothers included in the clinical assessments, two participants were not eligible for MRI scanning for medical reasons, two participants refused participation in an MRI scan, three participants were excluded due to excessive movement in the scanner (2) or technical problems during scanning (1). One participant was excluded due to abnormal brain anatomy (i.e., structural damage in prefrontal and occipital areas) that resulted from traumatic brain injury and consequent neurosurgery following a motor-vehicle accident during childhood. This resulted in 37 participants (16 mothers with IPV-PTSD, 6 subthreshold, and 15 non-PTSD controls) being included in the analysis of fMRI data.

#### Acquisition of Saliva and Extraction of DNA

In preparation for saliva sampling, participants were told not to eat or drink for 1 h prior to their parent–child lab visit as described above. The samples were taken via a Salivette® cotton swab (Sarstedt, reference number 51.1534, www.sarstedt.com) which participants were asked to keep in their mouth for 3 min. The cotton was then placed in a labeled plastic tube and frozen at -30°C until extraction. Saliva used for DNA extraction was drawn from the first saliva sample that had been taken prior to the mother–child interaction procedure whenever possible. DNA extracted from saliva (Cao-Lei et al., 2014; Weder et al., 2014) as well as buccal cells (Lowe et al., 2013) has been only recently found to be reliably comparable to that extracted from blood cells with particular reference to peripheral measurement of methylation of the *NR3C1* gene. To confirm this relationship, we compared blood and saliva samples for mean total percentage of *NR3C1* methylation in 15 pregnant women who gave informed consent for blood and saliva sampling in a concurrently running study by our research lab, and using the same methods for sampling and extraction as described below. The correlation of the blood lymphocyte and saliva-derived values indeed showed a robust, positive correlation (*r* = 0.60, *p* = 0.02; Sancho Rossignol et al., 2014). Genomic DNA was extracted from the Salivette® cotton swab using the DNA extraction kit produced by GE Healthcare (RPN8501), the quantity of DNA was assessed with Qubit (The Qubit® 2.0 Fluorometer, Invitrogen) and the quality was verified on gel electrophoresis.

#### *NR3C1* Methylation Status

The extracted DNA was then treated with sodium bisulfite in order to convert unmethylated cytosine residues to uracil using EpiTect Bisulfite Kit (Qiagen, CA, USA) according to the manufacturer’s protocol. The converted DNA was eluted in 20 μl of Elution Buffer (10 mM Tris-HCl, pH 8.5). Two microliter of the post bisulfite-treated DNA were used for subsequent PCR amplification.

The PCR amplifications aimed at pyrosequencing were performed starting from 100 to 140 ng of bisulfite-treated DNA. The PCR conditions were 94°C for 15 min, followed by 50 cycles of 94°C, 30 s, 52°C, 30 s, 72°C, 40 s, and by a 72°C 10 min final extension step.

The sequence of the oligonucleotides, within *NR3C1* (GenBank #AY436590) is the following: NR3HumF: 5′-TTTGAAGTTTTTTTAGAGGG-3′ and NR3HumR: 5′-biotin-7-CCCCCAACTCCCCAAAAA-3′ (adapted from Oberlander et al., 2008). Amplification resulted in a 403 bp fragment (position-3485 to -3082). The reactions were performed with a PCR reaction mixture (total volume 16 μl) containing oligonucleotides at 0.5 mM concentration and 7.5 μl of HotStarTaq Master Mix (Qiagen, CA, USA). The biotinylated PCR products were purified using streptavidin-sepharose beads (Amersham) and sequenced using the PSQ 96 Gold reagent kit (Biotage AB, Uppsala, Sweden) with the following primer: NR3HumS1: 5′-GAGTGGGTTTGGAGT-3′.

The degree of methylation at each CpG site was determined automatically by the Pyro Q-CpG Software using the C over T pics intensities that were produced at the 13 different CpG sites (Biotage AB, Uppsala, Sweden). The CpG3 and CpG4 are located within the binding motif for NGFI-A (Oberlander et al., 2008) and corresponds to the numerated CpG37 and CpG38 in another study (McGowan et al., 2009). The assay was performed in triplicate. We controlled for the quality of these data by analyzing five different human methylated standards (0, 25, 50, 75, and 100%) deriving from the commercial unmethylated (0%) and methylated (100%) genomic DNA products (EpiTect PCR Control DNA, Qiagen). Control samples that were 0 and 100% methylated were bisulfite converted independently. And each clinical sample and its replicates were also converted independently. Pearson correlations of the theoretical methylation percentages with the observed methylation percentages were significant for all 13 CpG sites tested (Supplementary Figure S1).

### Data Analysis

Differences between IPV-PTSD mothers and non-PTSD controls were analyzed with Mann–Whitney *U* tests for continuous variables (e.g., child age) and chi-square tests for categorical variables (e.g., mother’s physical abuse as a child). Associations between *NR3c1* methylation and nine continuous measures (e.g., maternal PTSD severity, parenting stress, degree of maternal sensitivity) were tested using Spearman correlations (*r*_s_) whenever at least one variable was not normally distributed and Pearson correlations (*r*) in all other instances. The only variables that met the normality assumption among these nine were the mean percentage of *NR3c1* methylation (Shapiro–Wilk statistic = 0.979; *p* = 0.505) and the Parenting Stress Index score (Shapiro–Wilk statistic = 0.977, *p* = 505). The Bonferroni correction was applied to adjust for multiple tests. In line with our *a priori* hypotheses, two different linear regression models were used to explore the combined effects of maternal PTSD symptom severity and mean total percentage of *NR3C1* methylation on parenting stress, as well as maternal PTSD symptom severity, reported parenting stress, and maternal *NR3c1* mean methylation on child cooperativeness. All analyses were performed using SPSS Versions 19 and 22 (IBM, Armonk NY, USA). Significance level was set at 0.05 (two-tailed tests).

#### Procedures Specific to fMRI Data

Preprocessing of the acquired Images is described in the Supplementary Materials.

In first level analysis, we produced a contrast between the average neural activity in response to separation among all children as compared to scenes of play. In second level analysis we then applied Pearson correlations to examine the associations between this contrast and mean NR3C1 methylation within a whole-brain analysis (Bogdan et al., 2013).

A cluster-extent based thresholding approach was used to correct for multiple comparisons created by the high number of voxels analyzed. A Monte Carlo simulation using Slotnik’s method (Slotnick et al., 2003) with 10,000 iterations indicated that a false–positive probability of 0.05 was achieved when applying the condition that each regional cluster must include at least 27 contiguous voxels (3mm^∗^3mm^∗^3mm) with an uncorrected *p*-value of <0.005.

For our whole-brain analysis, the threshold of significance was thus defined as an uncorrected *p* < 0.005 with at least 27 contiguous voxels necessary to constitute a significant finding.

#### Procedures Specific to Epigenetics

For variables and brain activity clusters that were significantly correlated to the mean percentage of methylation of *NR3C1*, we performed additional *post hoc* tests for associations with percentages of methylation at each of the 13 CpG sites. Non-parametric Spearman correlation coefficients were used, because the methylation of the individual CpGs was not normally distributed given that many participants had no methylation at a given CpG site (all 13 Shapiro–Wilk statistics <0.856, *p* < 0.001).

## Results

### Characteristics of Participants

Comparison of IPV-PTSD mothers (*n* = 28) and non-PTSD mothers (*n* = 17) indicated no group differences for maternal age, child age, or gender, SES, and maternal drug and alcohol abuse history (see **Table 1A**). Differences between groups were significant in terms of IPV trauma, psychopathology, parenting stress, and maternal behavior (**Table 1B**). IPV-PTSD mothers had more severe current and lifetime PTSD symptoms (*p* < 0.001). They were much more likely to have been physically abused (*p* = 0.006) and exposed to domestic violence as children (*p* = 0.015), but not sexually abused (*p* = 0.163) as children under the age of 16. However, even within the non-PTSD control group, 29% of mothers had been physically abused as children and 35% had experienced IPV as adults, although not generally at the hands of their partner. In keeping with the fact that recruitment efforts were in part organized around domestic violence agencies and related services, 82% of IPV-PTSD vs. 0% of non-PTSD mothers experienced partner violence as adults and all mothers with IPV-PTSD had experienced at least one type of violence as adults. As **Table 1B** shows, the IPV-PTSD group had experienced more severe IPV as adults than non-PTSD controls on the Conflict Tactics Scale (*p* = 0.004). IPV-PTSD mothers were somewhat more stressed as parents on the Parenting Stress Index, even though statistical significance was not reached (*p* = 0.091); and they were significantly less sensitive during play with their children (*p* = 0.011).

**Table 1A:** Differences between mothers suffering from Interpersonal violence-related posttraumatic stress disorder (IPV-PTSD) and non-PTSD controls.

	IPV-PTSD (*n* = 28) Median (SD)	Controls (*n* = 17) Mean (SD)	Mann–Whitney test *z*-value/Pearson chi-square	*p*
Maternal age (in years)	35.1 (5.8)	35.5 (5.5)	-0.29	0.77
Child age (in months)	27.0 (8.6)	28.2 (8.7)	-0.44	0.66
Child gender (percentage of boys)	50%	59%	0.56	0.33
Socio-economic status (SES; low values indicate higher status)	5.2 (2.1)	4.4 (1.9)	-1.22	0.22
Maternal drug and alcohol abuse history	24%	21%	0.053	0.82

The current PTSD symptom severity was measured with the Post-traumatic Symptom Checklist -Short Version (PCL-S). The lifetime PTSD symptom severity was measured with the Clinician Administered PTSD Scale. Parenting stress was measured with the Parenting Stress Index—Short Form (PSI-SF), and partner aggression was measured with the Conflict Tactics Scale (CTS). SD are shown in parentheses where applicable.

**Table 1B:** Socio-demographic differences between mothers suffering from IPV-PTSD and non-PTSD controls.

	IPV-PTSD (*n* = 28) Mean (SD)	Controls (*n* = 17) Mean (SD)	Mann-Whitney test *z*-value/Pearson chi-square	*P*
Current PTSD symptom severity	40.6 (10.3)	16.4 (0.3)	5.63	<0.001
Lifetime PTSD symptom severity	85.4 (19.5)	19.0 (6.3)	5.64	<0.001
Physical abuse as a child	71%	29%	7.56	0.006
Sexual abuse as a child	21%	6%	1.94	0.163
Exposed to domestic violence as a child	61%	24%	5.88	0.015
Experienced sexual and/or physical assault by partner as an adult	82%	0%	28.56	<0.001
Experienced sexual and/or physical assault by non-partner as an adult	82%	35%	10.13	0.001
Any kind of interpersonal violence as a child (experienced physical or sexual abuse and/or exposed to domestic violence)	89%	47%	9.64	0.002
Severity of current/last partner physical aggression	4.5 (8.8)	0.0 (0.0)	2.85	0.004
Parenting stress	42.8 (15.4)	35.0 (14.2)	1.69	0.091
Maternal sensitivity	5.0 (1.3)	6.1 (1.0)	2.56	0.011
*NR3c1* methylation (%)	3.8 (1.8)	5.3 (2.0)	2.26	0.013

SD are shown in parentheses.

### *NR3C1* Methylation

As indicated in **Table 1B**, IPV-PTSD mothers were characterized by a significantly lower mean percentage of methylation of the *NR3C1* gene.

Associations of *NR3C1* gene methylation with continuous measures are shown in **Table 2**. Mean percentage of methylation was significantly and negatively correlated with severity of maternal exposure to child physical abuse, maternal current PTSD severity and parenting stress, but not maternal sensitivity. In order to investigate more precisely the negative correlation between parenting stress and *NR3C1* methylation, we performed correlations for each subgroup of participants. The negative correlation between parenting stress and *NR3C1* methylation was primarily driven by maternal IPV-PTSD (*n* = 20, *r* = -0.529, *p* = 0.017) and subthreshold (*n* = 7, *r* = -0.764, *p* = 0.046) groups, rather than by the HC (*n* = 17, *r* = 0.060, *p* = 0.820). Mean percentage of methylation at CpGs sites 3, 4, 5, and 11 was correlated to parenting stress (CpG3: *r*_s_ = -0.291, *p* = 0.06; CpG4: *r*_s_ = -0.266, *p* = 0.08; CpG5: *r*_s_ = -0.358, *p* = 0.02; CpG11: *r*_s_ = -0.522, *p* < 0.001) as shown in Supplementary Table S1. Only the association between CpG 11 and parenting stress withstood Bonferroni corrections for multiple tests. No specific CpG was correlated to IPV-PTSD symptom severity.

**Table 2:** Correlations with *NR3C1* mean methylation.

	Correlation coefficient with *NR3c1* mean methylation	Significance (*p*)
**Socio-economic status**	***r*_**s**_ = -0.361**	**0.016**
**Physical abuse as a child (BPSAQ, Brief Physical and Sexual Abuse Questionnaire)**	***r*_**s**_ = -0.347**	**0.020**
Sexual abuse as a child (BPSAQ)	*r*_**s**_ = 0.094	0.560
Severity of partner physical aggression (CTS)	*r*_s_ = -0.202	0.194
**Current PTSD symptom severity (PCL-S)**	***r*_**s**_ = -0.489**	**0.002^a^**
**Parenting Stress (PSI-SF)**	*****r*** = -0.436**	**0.003^**a**^**
Maternal Sensitivity (CARE-Index)	*r*_s_ = 0.265	0.078
**Child Cooperativeness (CARE-Index)**	***r*_**s**_ = 0.375**	**0.011**

Associations of NR3C1 gene methylation with socio-economic and clinical measures (*n* = 45), *r* = Pearson correlations (only applied if both variables passed normality tests), *r*_s_ = Spearman correlation, ^a^*p* < 0.05 after Bonferroni correction for multiple tests.

Bolded values represent significant findings.

As detailed in Supplementary Table S2, we next explored via regression analysis if maternal *NR3C1* methylation would remain significantly associated with parenting stress (β = -0.44, *p* = 0.003) after controlling for maternal IPV-PTSD symptoms. Indeed, this association did remain significant (β = -0.40, *p* = 0.006). We also tested whether the association of maternal *NR3C1* methylation with observed child cooperativeness in play (β = 0.39, *p* = 0.009) would remain significant after controlling for maternal IPV-PTSD symptoms and parenting stress. This association, however, no longer remained significant after controlling for those two variables (β = 0.19, *p* = 0.265).

### fMRI Results

**FIGURE 1:**
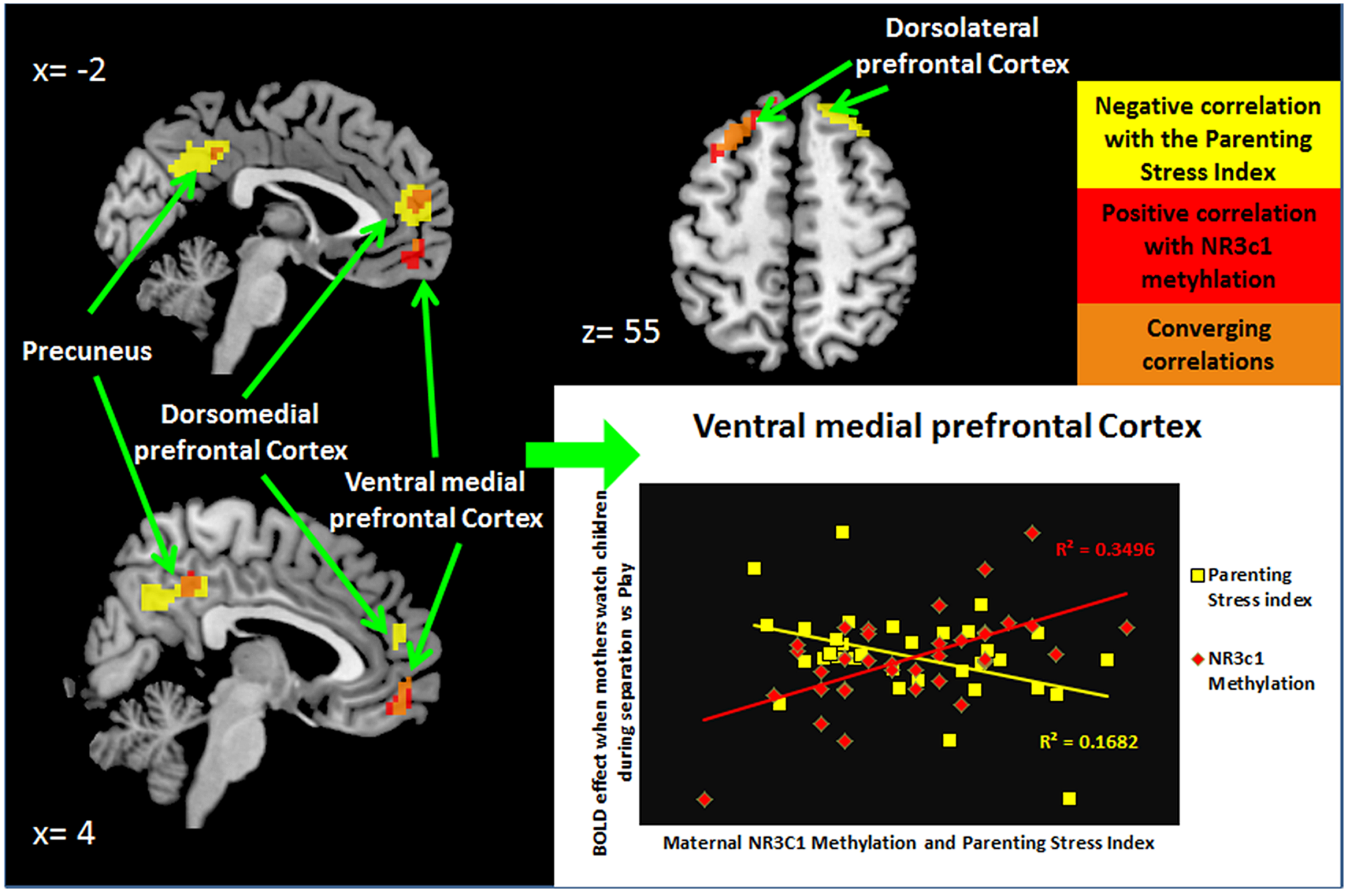
***NR3C1* methylation correlated to functional brain activity when mothers see children during separation compared to play**.

We first examined the relationship of maternal PTSD severity and parenting stress to neural activity to test if affected brain-areas associated with *NR3C1* methylation would also be associated with observable clinically relevant dysfunction (**Table 3** and Supplementary Table S3). Maternal PTSD severity correlated negatively with activity in the mPFC and the dorsolateral prefrontal cortex (dlPFC) as shown in **Table 3**. As shown in **Table 3** and **Figure 1**, the degree of parenting stress correlated negatively with neural activity in the ventromedial prefrontal cortex (vmPFC; *r* = -0.410, *p* = 0.013) and the dorsomedial prefrontal cortex (dmPFC; *r* = -0.546, *p* = 0.001).

**Table 3:** Mean percentage of methylation of *NR3C1* correlated with BOLD activity when mothers watch separation vs. play scenes.

Cluster size	MNI location of the peak voxel			Regions comprised in this cluster	Correlation of cluster activity with *NR3C1*				
	*x*	*y*	*z*		*r*-value within: IPV-PTSD Subthreshold HC	*p*-value within: IPV-PTSD Subthreshold HC	Correlation of cluster activation with *NR3c1* methylation	Correlation of cluster activation with parenting stress	Correlation of cluster activation with current PTSD symptom severity
199	12	53	-11	vmPFC, OFC, right gyrus rectus	0.529 0.925 0.219	0.043 0.003 0.432	*r* = 0.591 *p* < 0.001	*r* = -0.410 *p* = 0.013	*r*_s_ = -0.368 *p* = 0.012
127	-24	29	55	Left dlPFC	0.619 0.826 0.259	0.014 0.022 0.352	*r* = 0.548 *p* < 0.001	*r* = -0.462 *p* = 0.005	*r*_s_ = -0.497 *p* = 0.002
32	-51	11	43	Left dlPFC, cortex left precentral gyrus	0.647 0.899 0.302	0.009 0.006 0.275	*r* = 0.552 *p* < 0.001	*r* = -0.246 *p* = 0.148	*r*_s_ = -0.399 *p* = 0.014
34	-6	53	13	dmPFC	0.487 0.922 0.180	0.066 0.003 0.522	*r* = 0.495 *p* = 0.002	*r* = -0.546 *p* = 0.001	*r*_s_ = -0.356 *p* = 0.030
30	15	-25	19	Thalamus	0.359 -0.132 0.662	0.189 0.777 0.007	*r* = 0.493 *p* = 0.002	*r* = -0.308 *p* = 0.068	*r*_s_ = -0.370 *p* = 0.024
64	-27	-49	49	Left precuneus, posterior cingulate cortex	0.450 0.634 0.587	0.093 0.126 0.021	*r* = 0.527 *p* = 0.001	*r* = -0.438 *p* = 0.008	*r*_s_ = -0.392 *p* = 0.016

dlPFC, dorsolateral Prefrontal Cortex; dmPFC, dorsomedial Prefrontal Cortex; HC, healthy controls; IPV-PTSD, mothers with interpersonal violence related post traumatic stress disorder; OFC, orbitofrontal cortex; vmPFC, ventromedial Prefrontal Cortex, *r* = Pearson correlation, *r*_s_ = Spearman correlation, *p* = significance value.

**FIGURE 2:**
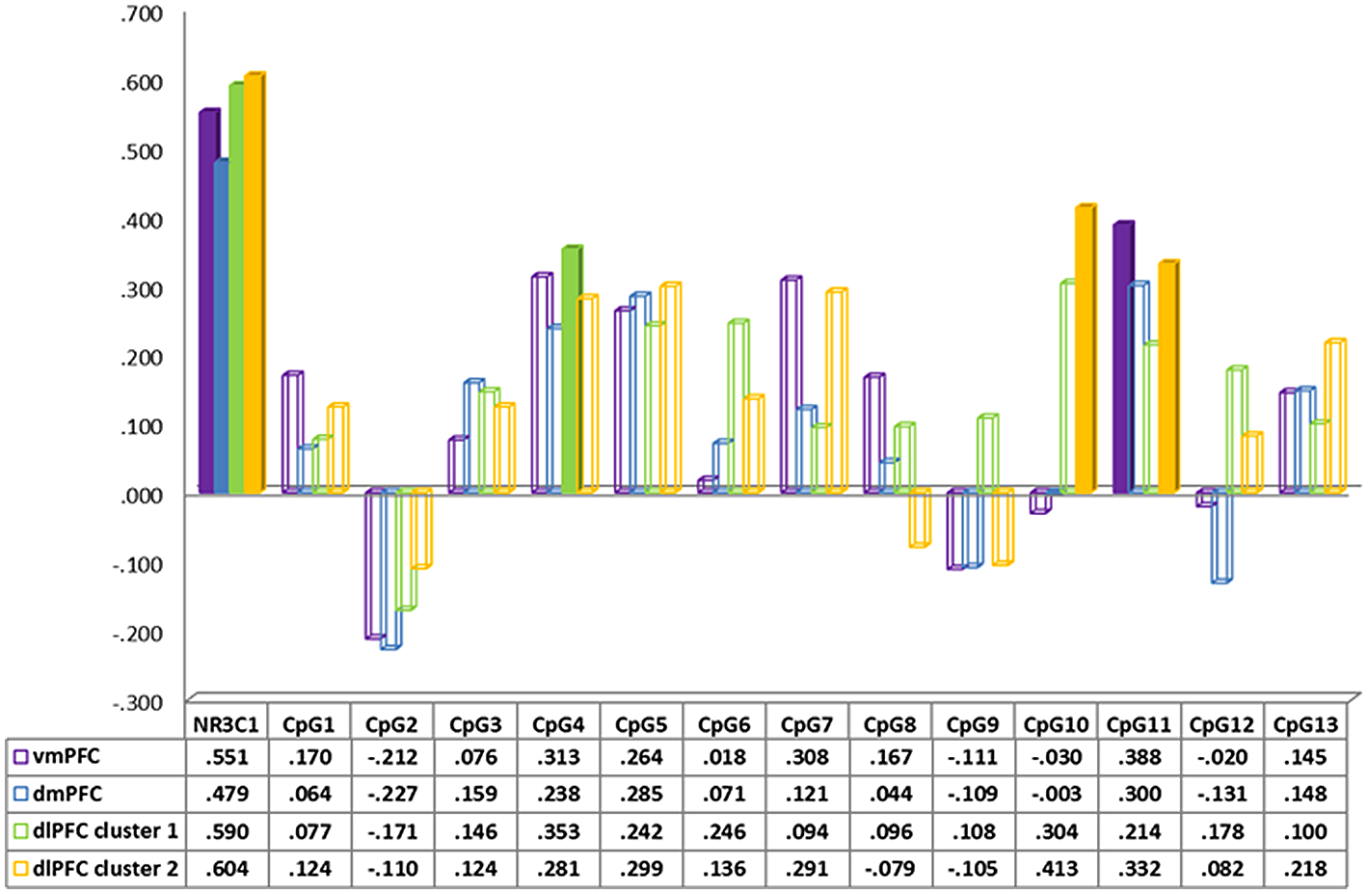
**Spearman correlation between neural activity in prefrontal cortex regions during separation vs. play and CpG methylation of the *NR3c1***.

We next examined the relationship of percentage of methylation of *NR3C1* to maternal clinically—relevant neural activity in response to mothers’ viewing of their own child and unfamiliar children in separation versus play. As shown in **Figure 2** and **Table 3**, the mean percentage of methylation of the *NR3C1* gene positively correlated with activity in the vmPFC, dmPFC, and left dlPFC, as well as the precuneus and thalamus. Thus, all three of the brain areas that showed significant relationships to parenting stress were also significantly related to the percentage of methylation of the *NR3C1* gene peripherally, and in corresponding directions of effect (i.e., less dmPFC and vmPFC activation with greater maternal PTSD severity and parenting stress, and less methylation of the *NR3C1* gene).

In order to understand better the relative importance of different factors, we then extracted the average activity of the clusters found in the whole brain analysis of the correlation with *NR3C1*. For each cluster, we performed a regression analysis in which neural activity was the dependent variable and *NR3C1* methylation and reported parenting stress were the independent variables. Within this model, *NR3C1* methylation but not parenting stress was a significant predictor of neural activity in the dlPFC and vmPFC clusters. Parenting stress but not *NR3C1* methylation was a significant predictor in the dmPFC cluster (see Supplementary Table S3).

In order to better understand the origins of these effects, we performed *post hoc* Spearman correlations of the mean percentage of (1) methylation at each of the 13 tested CpG sites of the NR3C1 gene and (2) neural activity in the different brain-region clusters (**Figure 2**, together with Supplementary Figures S2 and S3). None of these associations withstood Bonferroni corrections for multiple tests. Mean methylation at CpG4 was correlated with neural activity in the vmPFC (*r*_s_ = 0.313, *p* = 0.03), and the dlPFC (*r*_s_ = 0.313, *p* = 0.06). In addition, mean methylation of CpG5 was correlated with neural activity in the dlPFC (*r*_s_ = 0.299, *p* = 0.07). Finally mean methylation at CpG11 was correlated with neural activity in the vmPFC (*r*_s_ = 0.388, *p* = 0.02) and the dlPFC (*r*_s_ = 0.332, *p* = 0.01).

## Discussion

Results of this study have demonstrated important, convergent associations between the mean percentage of methylation of the promoter region of the *NR3C1* gene and the following key variables: maternal IPV-PTSD, parenting stress, and neural activity in cortical regions that are implicated in emotion regulation; namely, the vmPFC, dmPFC, and dlPFC. These areas are similarly associated with parenting stress and maternal PTSD symptom severity in response to stressful silent video stimuli of a routine relational stressor (i.e., mother–child separation) versus less stressful silent video stimuli (i.e., mother–child play).

Lower neural activity in the prefrontal cortex and greater parenting stress were both associated with a lower mean percentage of methylation overall. Given sample-size limitations and the number of comparisons necessary to examine all 13 CpG sites, it was not surprising that none of the associations to CpG sites with neural activity remained significant after Bonferroni correction for multiple comparisons. About the contribution of the differing CpGs, we can thus only speculate with the severe limitation of uncorrected correlations. Those CpG correlations suggested that several CpG sites including CpG4, CpG5, and CpG11 might be associated to neural activity in several key prefrontal regions such as vmPFC and dlPFC within a larger sample and should be studied further in the future. Of note, CpG4 would be functionally relevant since it is located within the binding site of the nerve growth factor induced protein A (NGFI-A; Oberlander et al., 2008; McGowan et al., 2009). NGFI-A binding leads to an increase in *NR3C1* expression and methylation decreases expression of *NR3C1* (McGowan et al., 2009).

While methylation of the *NR3C1* gene was not significantly associated with maternal sensitivity, the overall mean percentage of methylation of *NR3C1* (as well as at the CpG4 and 11 sites) were negatively associated with parenting stress and positively associated with observed child cooperativeness during play. The overall mean percentage of methylation of *NR3C1* together with maternal current PTSD symptom severity accounted for 22.5% of the variance of parenting stress.

The present study is thus the first to our knowledge to link findings within different psychobiological domains (i.e., post-traumatic stress, relational, or “parenting” stress, epigenetics linked to the HPA-axis stress response, and neural activity) in support of the notion that *NR3C1* epigenetic signatures that are characterized by low-methylation might denote risk to the early mother–child relationship. Risk to the early mother–child relationship was marked by increased parenting stress, which in turn might increase the risk for developmental psychopathology in her child. IPV-PTSD mothers with lower *NR3C1* methylation had a greater tendency to report more subjective parenting stress. Increased parenting stress was significantly associated with less maternal vmPFC and dmPFC on fMRI. A subsequent regression analysis (see Supplementary Table S3) suggested that neural activity in several regions (dlPFC, vmPFC, OFC) was at least as well predicted by *NR3C1* methylation as by reported parenting stress, suggesting a link between HPA axis physiology and brain processing of stimuli triggering parenting stress. These results echo findings by the corresponding author from a previous study with a different sample (Schechter et al., 2012). In that study, IPV-PTSD mothers reported experiencing significantly more stress upon viewing video excerpts of parent–child separation versus play which corresponded to less medial prefrontal cortical activity than among non-PTSD controls in response to the same stimuli.

The association in the present paper between corticolimbic dysregulation and *NR3C1* methylation supports the hypothesis that a maternal endophenotype which carries a low mean percentage of *NR3C1* methylation is likely to be associated with parenting stress and that parenting stress, coupled with maternal IPV-PTSD likely adversely impacts the quality of maternal behavior (i.e., maternal sensitivity and responsiveness to child bids for joint attention; Schechter et al., 2010). Difficulty in parental regulation of normative aggression in early childhood has been shown to be a potent risk factor for subsequent conduct disorder (Cote et al., 2006). At least one prior study has noted dysregulated aggression as well as anxiety, avoidance, and hypervigilance to danger in the child, as being associated with greater maternal IPV-PTSD severity (Schechter et al., 2007). Further study is needed to see if this tendency is greater among children whose mothers have a particular epigenetic signature.

In the present study, the severity of IPV-PTSD was linked not only to the severity of adult partner violence, but also to the severity of mothers’ childhood exposure to physical abuse. Given that the methylation of the *NR3C1* gene is known to be a biological signature for early life stress (McGowan et al., 2009), we cannot exclude that maternal history of childhood physical abuse is driving the convergence of findings. The complexity of the mothers’ history, involving multiple exposures and the development of PTSD symptoms, given our limited sample size, does not allow us to tease apart these different life-event and psychopathology variables. We can say, however, that the present study did not replicate the direction of effect of prior studies that looked at retrospective assessment of early life adversity using a self-report questionnaire rather than a clinical interview to review early life events (Perroud et al., 2011) or in the post-mortem study that did use a clinical interview to review life events but within a limited sample of male subjects who had committed suicide and who were described as having major depression as a primary diagnosis (Dumais et al., 2005; McGowan et al., 2009). In the Perroud et al. (2011) study, childhood exposure to sexual and emotional abuse were associated with increased mean percentage of methylation on the *NR3C1* gene’s promoter region, thus a positive association, among women primarily with borderline personality disorder, many with comorbid major depression.

A negative association between percentage of methylation on the *NR3C1* gene’s promoter region and post-traumatic stress disorder has been established in prior studies of combat veterans who clearly experienced violence but whose early life-histories are unclear (Yehuda et al., 2014b). Our finding that the severity of maternal PTSD was inversely correlated with the degree of methylation of the *NR3C1* promoter region suggests that the effect in either direction is a biological signature of early life stress, but that the direction of effect is most likely dependent on how the exposure is processed by the organism. In other words, the psychophysiology of PTSD as a particular reaction to early adverse life events plays an important role in the direction of effect.

Socio-economic status correlated with methylation of the *NR3C1*. It is beyond the scope of this study to determine whether we might expect SES to be serving as a marker for more severe, earlier onset, and more chronic histories of adverse life-events linked with lower SES, or if, alternatively, the epigenetic signature is tied to lower SES as a marker of impaired functioning or another confounding variable.

### Limitations

One methodological limitation of this study is that hydroxy-methylation of cytosine was not discriminated from methylation of cytosine in bisulfite-pyrosequencing (Huang et al., 2010).

An important limitation of this study is that by its cross-sectional design, we could only measure associations rather than prediction and direction of effects. For example, it could be the case as has been postulated (Engel et al., 2005) that low percentage of methylation *NR3C1* epigenetic signature could represent an effect of fetal programming and be a risk factor for the development of PTSD rather than an associated feature or effect of PTSD. Similarly, as noted above, we cannot tease apart whether early and chronic exposure to physical abuse and domestic violence alone might be necessary and sufficient to generate the epigenetic signature if occurring during a sensitive window of development, or if while necessary, it remains insufficient, and the development of PTSD is necessary.

Finally, sample size in the present study provided limited statistical power to study the interplay between numerous factors such as relationships to the CpG sites without the potential for Type I error. Structural equation modeling was thus not feasible, yet would be a useful method for future studies with a larger sample size.

### Clinical Implications

The present paper is consistent with prior clinical research findings that mothers with IPV-PTSD, who often suffer from early onset, repetitive, and chronic exposures to violence, report greater stress in parenting their very young children. This parenting stress and the maternal PTSD that contributes to it, have a negative effect on maternal sensitivity. This paper has shown that there are endophenotypic differences or biological signatures both at the level of epigenetics, namely methylation of the glucocorticoid receptor *NR3C1* and at the level of neural activity, principally decreased activity of the vmPFC, dmPFC, and dlPFC in response to viewing mother–child separation versus play, which serve as markers for parenting stress among the traumatized mothers who participated. These biomarkers will be tested in ongoing research to see if they can serve as clinical indicators of risk and of potential change with intervention.

## Conflict of Interest Statement

The authors declare that the research was conducted in the absence of any commercial or financial relationships that could be construed as a potential conflict of interest.
