# Supplementary material for: Methylation of NR3C1 is related to maternal PTSD, parenting stress and maternal medial prefrontal cortical activity in response to child separation among mothers with histories of violence exposure
Source: Front Psychol. 2015 May 29;6:690. doi: 10.3389/fpsyg.2015.00690 (PMC4447998; doi:10.3389/fpsyg.2015.00690)
Supplement: Supplementary file 1 [file Table_1.PDF]

Supplementary Materials

fMRI Image Acquisition and Pre-Processing

Scanning took place in with a scanner provided by the Geneva branch of the Center for Biomedical Imaging (CIBM) of the Geneva-Lausanne Universities, the EPFL and the Geneva-Lausanne University Hospitals. All images were acquired on a Siemens 3 Tesla TrioTim scanner using a 12 channel receiver. After a three plane localizing image, a T1-weighted, Spoiled Gradient Recall image was acquired in the sagittal plane to prescribe the location of the anterior commissure–posterior commissure (AC/PC) line. Axial functional images positioned parallel to the AC/PC line were obtained using a T2*-weighted gradient-recalled single-shot echo planar pulse sequence with TR=2100ms, TE=30ms, 80º flip angle, 25.6x25.6cm^2 field of view, and a 64x64 voxel slice matrix. We acquired 36 slices of 3.5 mm thickness with a spacing of 0.5 mm to provide an effective resolution of 4 x 4 x 3.5 mm^3. Slices were acquired in interleaved order and spanned the entire brain. The functional echo planar images were preprocessed and statistically analyzed using batch programming based on SPM8 under MATLABR2012a. Prior to analysis, images were visually inspected for major artifacts and signal dropout. Images were realigned to the middle slice of each scan. After motion correction, functional images were co-registered with the anatomical image of the same participant prior to being spatially normalized and reformatted into a 3x3x3 mm resolution space. Finally, normalized images were spatially smoothed using a Gaussian filter with a full-width half-maximum of 8mm.

fMRI Thresholding

A cluster-extent based thresholding approach was used to correct for multiple comparisons. A Monte Carlo simulation with 10,000 iterations indicated that a false positive probability of 0.05 was achieved when implementing the condition that a cluster of at least 27 contiguous voxels displays an effect with $p<.005$.

Supplementary Table 1

	NR3c1	CpG1	2	3	4	5	6	7	8	9	10	11	12	13
**Current PTSD (PCLS)**	**-.49*** **													
**Parenting Stress Index**	**-.45** **			-.29+	**.27+**	**-.36***						**-.52[a],*** **		

Psychological and behavioral variables correlated to CpGs (Spearman correlation coefficients, n = 45). Uncorrected for multiple tests: +p<.1, *p<0.05, **p<0.01, ***p<0.001 ; corrected for multiple tests according to Bonferroni: [a] p < 0.05

Supplementary table 2

	Parenting Stress Index			Child Cooperativeness		
Independent variables	Standardized beta	t	p	Standardized beta	t	p
Current maternal IPV-PTSD symptoms	0.19	1.37	0.18	-0.28	-1.73	0.091
Reported parenting stress				-0.10	-0.63	0.534
Maternal NR3c1 methylation	-0.40	-2.89	0.006	0.19	1.13	0.265
Overall model	R^2=0.22	F=5.96	0.005	R^2=0.46	F=3.56	0.022

Regressions of parenting stress with NR3c1 methylation and current IPV-PTSD symptoms as independent variables, and of observed child cooperativeness, with maternal parenting stress, maternal IPV-PTSD, maternal NR3c1 methylation as independent variables.

Supplemental Table 3

cluster size	T value of the peak voxel	significance of the peak voxel	r value of the peak voxel	MNI location of the local peak voxels x	y	z	Region	This cluster also includes	Regression model with NR3c1 and parenting stress NR3c1:	Parenting stress:
199	4.42	<.001	0.599	12	53	-11	Ventromedial Prefrontal Cortex		β = .496 p = .005	β = -.155 p = .352
	4.27	<.001	0.585	0	53	-17	Right Gyrus Rectus			
	3.36	0.001	0.494	30	32	-20	Right Orbitofrontal Cortex			
127	3.95	<.001	0.555	-24	29	55	Left dorsolateral prefrontal cortex		β =.406 p = .020	β = -.253 p = .136
	3.86	<.001	0.546	-15	41	52	Left dorsolateral prefrontal cortex			
	3.51	0.001	0.510	-30	38	46	Left dorsolateral prefrontal cortex			
32	4.53	<.001	0.608	-51	11	43	Left dorsolateral prefrontal cortex	Left Precentral Gyrus	β = .560 p = .002	β = .042 p = .807
	3.60	<.001	0.520	-45	17	49	Left dorsolateral prefrontal cortex			
30	3.58	0.001	0.518	15	-25	19	Thalamus		β = .491 p = .008	β = -.055 p = .752
	3.26	0.001	0.483	27	-31	16	Thalamus			
34	3.56	0.001	0.516	-6	53	13	dorsomedial Prefrontal Cortex		β = .266 p = .114	β = -.409 p = .018
64	3.54	0.001	0.514	-27	-49	49	Left Precuneus	Posterior Cingulate Cortex	β = .413 p = .019	β = -.225 p = .189
	3.24	0.001	0.480	3	-43	43	Precuneus			
	3.06	0.002	0.459	-12	-46	40	Left Precuneus			

Mean percentage of methylation of NR3C1 correlated with BOLD activity when mothers watch separation vs play scenes. Parenting stress, and percentage of methylation of NR3C1 were also included as independent variables of a regression model to predict neural activity.

Supplemental Figure 1

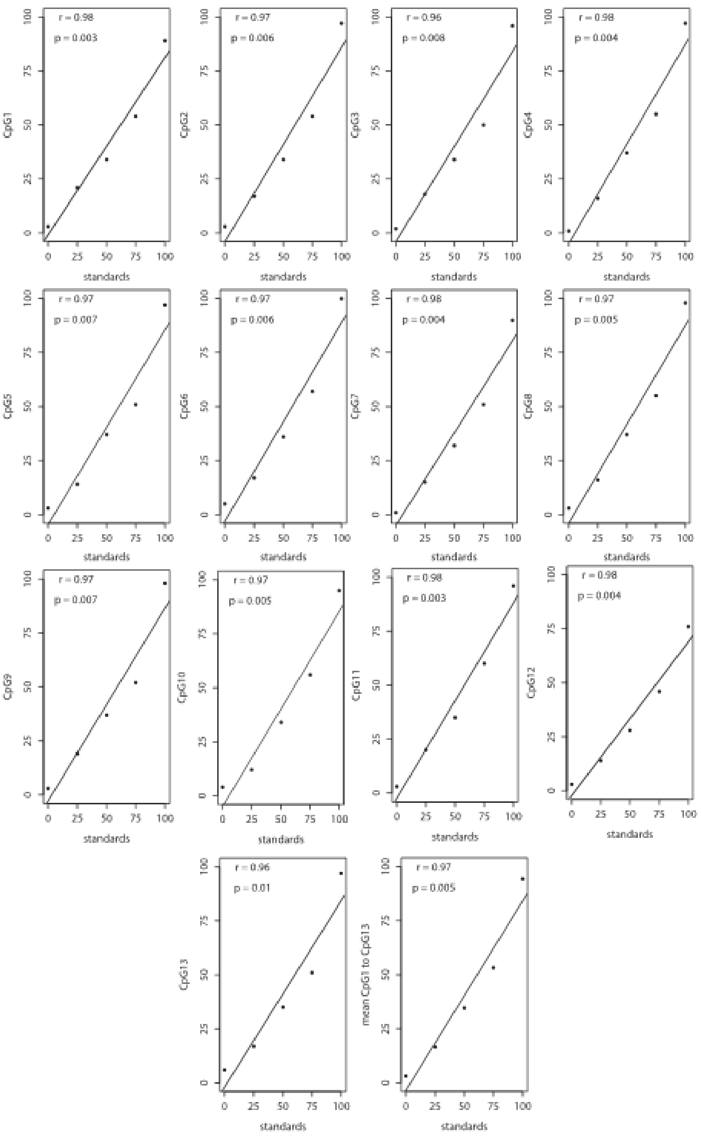

Control of the quality of the CpG methylation data by bisulfite-pyrosequencing, via analyzing five different human methylated standards (0%, 25%, 50%, 75%, 100%) deriving from the commercial unmethylated (0%) and methylated (100%) genomic DNA products (EpiTect PCR Control DNA, Qiagen). Pearson correlations of the theoretical methylation percentages with the observed methylation percentages were significant for all 13 CpG sites tested

Supplemental Figure 2

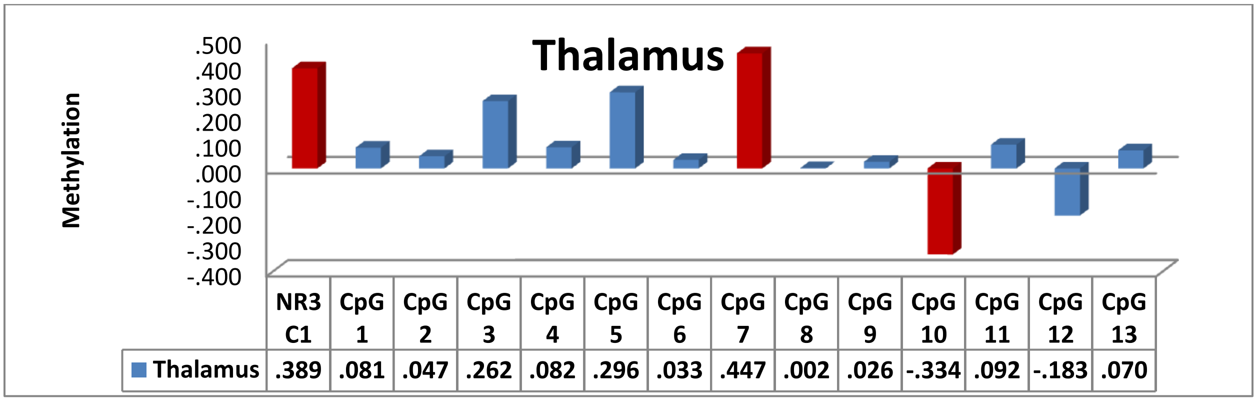

Supplemental Figure 2 shows Spearman correlations between the methylation of CpG sites of the NR3c1 gene, and brain activity in a significant cluster in the thalamus as measured by the blood oxygenation level dependent effect when 37 mothers watched children during separation vs play. Red bars are significant at $p<.05$. Blue bars are not significant.

Supplemental Figure 3

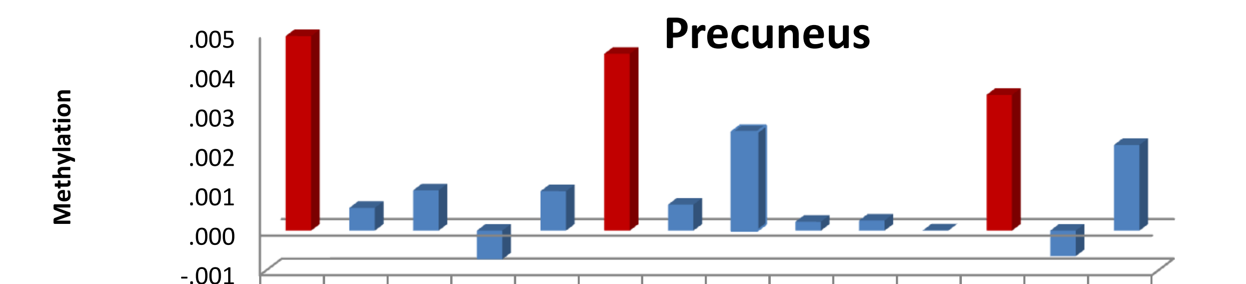

	NR3 C1	CpG 1	CpG 2	CpG 3	CpG 4	CpG 5	CpG 6	CpG 7	CpG 8	CpG 9	CpG 10	CpG 11	CpG 12	CpG 13
Precuneus	.493	.058	.102	-.072	.100	.448	.066	.250	.022	.026	.001	.344	-.065	.217

Supplemental Figure 3 shows Spearman correlations between the methylation of CpG sites of the NR3c1 gene, and brain activity in a significant cluster in the precuneus as measured by the blood oxygenation level dependent effect when 37 mothers watched children during separation vs play. Red bars are significant at p<.05. Blue bars are not significant.
